# Supplementary material for: Category Theoretic Analysis of Hierarchical Protein Materials and Social Networks
Source: PLoS One. 2011 Sep 8;6(9):e23911. doi: 10.1371/journal.pone.0023911 (PMC3169555; doi:10.1371/journal.pone.0023911)
Supplement: Figure S1 — Commutativity in the olog of the protein. In each of these eight diagrams, there are two paths from the upper left-hand box to the lower right-hand box. By stating that these diagrams are commutative, we are saying that these two paths are equivalent – given the same input they produce the same output. For example it is declared 30;39 = 32;35 : N→U, which means that starting with a pair (b,g) of building blocks serving as bricks and glue, one can obtain a building block in two ways, but either way the answer is the same: the brick. Similarly, 31;40 = 32;36 : N→U, which means that again starting with (b,g) we can again obtain a building block in two ways, but either way the answer will be the same: glue. An example of a non-commutative diagram found in the original olog is: 31;40 ≠ 30;39 : N→U. Starting with a pair (b,g), the path 31;40 produces its glue element whereas the path 30;39 produces its brick element. These facts are in some sense obvious, but to make ologs a rigorous system such facts must be recorded. (PDF) [file pone.0023911.s001.pdf]

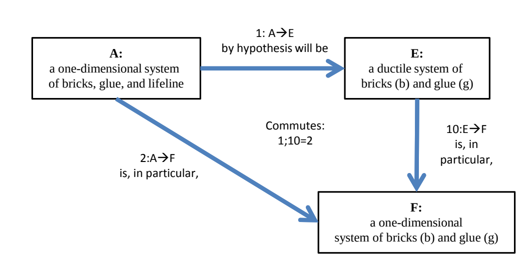

The conjecture that an arrow "**1:A→E**" exists such that this diagram commutes is the conjecture that a one-dimensional system of bricks, glue, and lifeline will always be ductile.

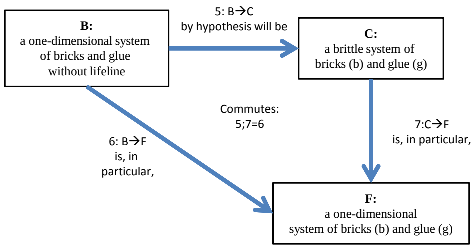

The conjecture that an arrow "5:**B→C**" exists such that this diagram commutes is the conjecture that a one-dimensional system of bricks and glue without lifeline will always be brittle.

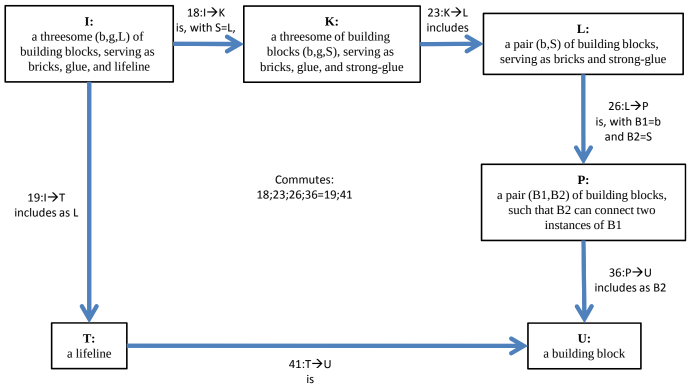

The commutativity of this diagram is a "check" on the meanings of the arrows: given a threesome (b,g,L) of building blocks, serving as bricks, glue, and lifeline, this diagram shows two different paths **I→U** to get a building block. Either way, one obtains the *same* building block, namely the lifeline L.

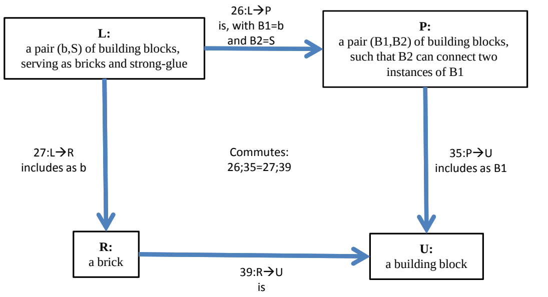

The commutativity of this diagram is a "check" on the meanings of the arrows: given a pair(b,S) of building blocks, serving as bricks and strong-glue, this diagram shows two different paths **L→U** to get a building block. Either way, one obtains the *same* building block, namely the strong-glue S.

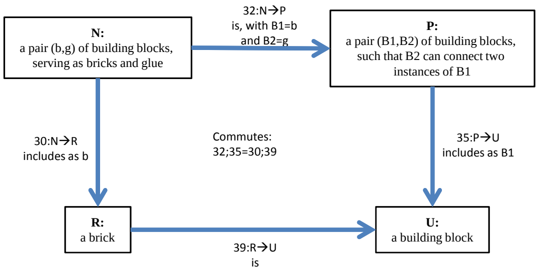

The commutativity of this diagram is a "check" on the meanings of the arrows: given a pair (b,g) of building blocks, serving as bricks and glue, this diagram shows two different paths **N→U** to get a building block. Either way, one obtains the *same* building block, namely the brick b.

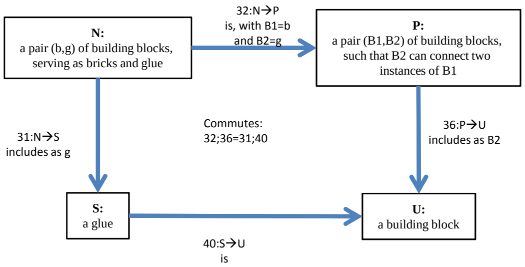

The commutativity of this diagram is a "check" on the meanings of the arrows: given a pair (b,g) of building blocks, serving as bricks and glue, this diagram shows two different paths **N→U** to get a building block. Either way, one obtains the *same* building block, namely the glue g.

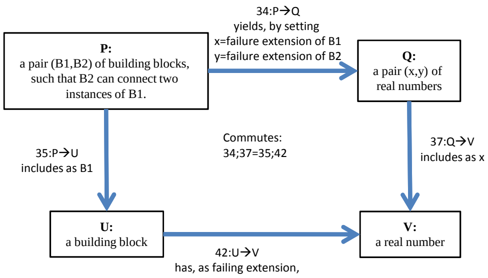

The commutativity of this diagram is a "check" on the meanings of the arrows: given a pair (B1,B2) of building blocks, such that B2 can connect two instances of B1, this diagram shows two different paths **P→V** to get a real number. Either way, one obtains the *same* real number, namely the failure extension of B1.

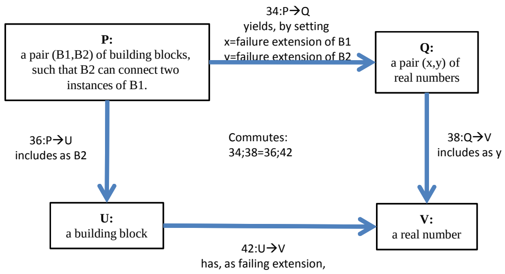

The commutativity of this diagram is a "check" on the meanings of the arrows: given a pair (B1,B2) of building blocks, such that B2 can connect two instances of B1, this diagram shows two different paths **P→V** to get a real number. Either way, one obtains the *same* real number, namely the failure extension of B2.
