# Supplementary material for: Category Theoretic Analysis of Hierarchical Protein Materials and Social Networks
Source: PLoS One. 2011 Sep 8;6(9):e23911. doi: 10.1371/journal.pone.0023911 (PMC3169555; doi:10.1371/journal.pone.0023911)
Supplement: Figure S2 — Fiber products in the olog of the protein. In each case, the upper left-hand box is the “fiber product” of the rest of the square. The property of being a fiber product defines the upper left-hand object: for example the notion of “one-dimensionality” in box A is defined for a system of bricks, glue, and lifeline by examining the structure of that system as a graph, and forcing that this graph is a chain graph (i.e. the elements are connected one to the next in a line). (PDF) [file pone.0023911.s002.pdf]

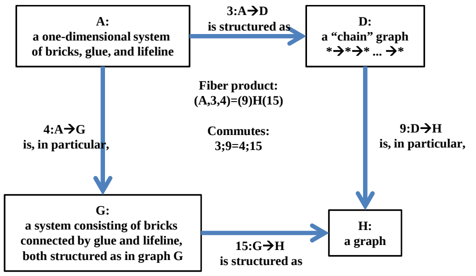

The fact that this square is a fiber product in effect defines (A) “a one-dimensional system of bricks, glue, and lifeline” to be a system consisting of bricks connected by glue and lifeline, both structured as in graph G, where G is a “chain” graph *→*→* ... →* .

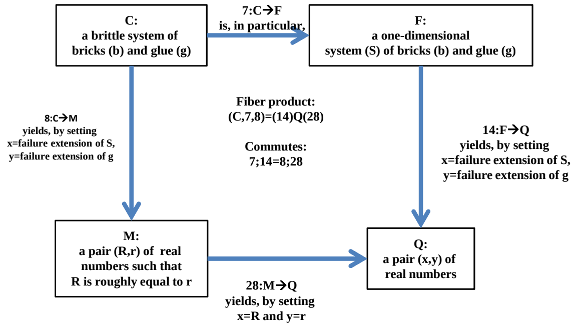

The fact that this square is a fiber product in effect defines (C) “a brittle system (S) of bricks (b) and glue (g)” to be a one-dimensional system of bricks (b) and glue (g) such that the failure extension of S is roughly equal to the failure extension of g.

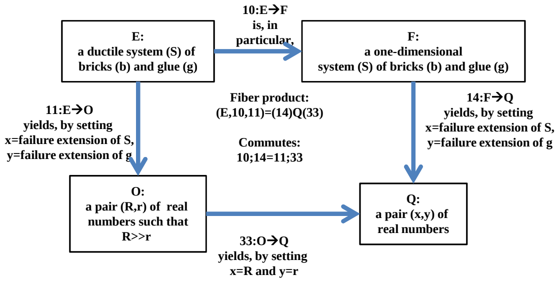

The fact that this square is a fiber product in effect defines (E) “a ductile system of bricks (b) and glue (g)” to be a one-dimensional system (S) of bricks (b) and glue (g) such that the failure extension of S is much greater than the failure extension of g.

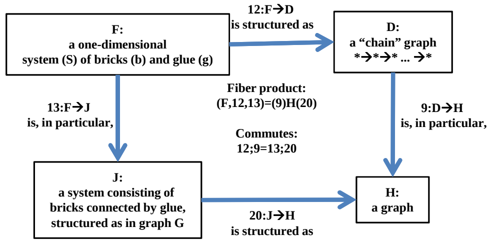

The fact that this square is a fiber product in effect defines (F) “a one-dimensional system (S) of bricks (b) and glue (g)” to be a system consisting of bricks connected by glue, structured as in graph G, where G is a “chain” graph *→*→* ... →* .

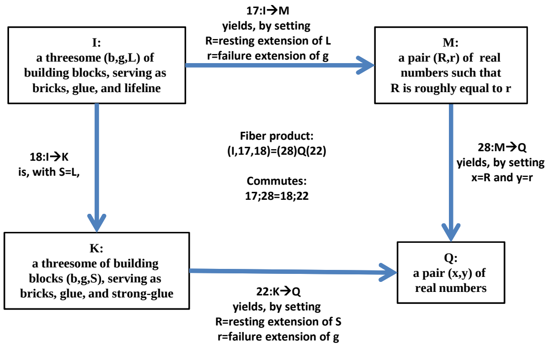

The fact that this square is a fiber product in effect defines (I) “a threesome (b,g,L) of building blocks, serving as bricks, glue, and lifeline” to be a threesome of building blocks (b,g,S), serving as bricks, glue, and strong-glue, such that the resting extension of the lifeline is roughly equal to the failure extension of the glue.

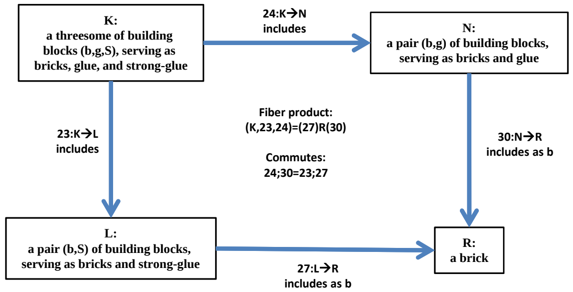

The fact that this square is a fiber product in effect defines (K) “a threesome (b,g,L) of building blocks, serving as bricks, glue, and strong-glue” to be a pair of building blocks serving as bricks and glue together with a pair of building blocks serving as bricks and strong-glue, where the bricks are the same in both cases.

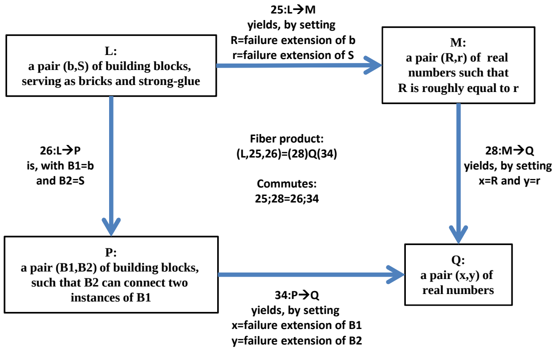

The fact that this square is a fiber product in effect defines (L) “a pair (b,S) of building blocks, serving as bricks and strong-glue” to be a pair of building blocks such that S can connect two instances of b and such that the failure extension of b is roughly equal to the failure extension of S.

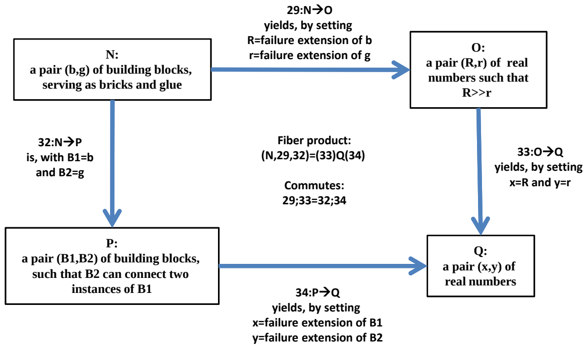

The fact that this square is a fiber product in effect defines (N) “a pair (b,g) of building blocks, serving as bricks and glue” to be a pair of building blocks such that g can connect two instances of b and such that the failure extension of b is much greater than the failure extension of g.
